# Deubiquitinase YOD1 suppresses tumor progression by stabilizing E3 ligase TRIM33 in head and neck squamous cell carcinoma

**DOI:** 10.1038/s41419-023-06035-0

**Published:** 2023-08-12

**Authors:** Yue Wu, Yuansheng Duan, Wei Han, Jiayan Cao, Beibei Ye, Peng Chen, Hong Li, Yuxuan Wang, Jin Liu, Yan Fang, Kai Yue, Yansheng Wu, Xudong Wang, Chao Jing

**Affiliations:** Department of Maxillofacial and Otorhinolaryngological Oncology, Tianjin Medical University Cancer Institute & Hospital, National Clinical Research Center for Cancer; Key Laboratory of Cancer Prevention and Therapy, Tianjin; Tianjin’s Clinical Research Center for Cancer, Tianjin, 300060 China

**Keywords:** Oral cancer, Metastasis, Tumour-suppressor proteins

## Abstract

Ubiquitination is a reversible process that not only controls protein synthesis and degradation, but also is essential for protein transport, localization and biological activity. Deubiquitinating enzyme (DUB) dysfunction leads to various diseases, including cancer. In this study, we aimed to explore the functions and mechanisms of crucial DUBs in head and neck squamous cell carcinoma (HNSCC). Based on bioinformatic analysis and immunohistochemistry detection, YOD1 was identified to be significantly downregulated in HNSCC specimens compared with adjacent normal tissues. Further analysis revealed that reduced YOD1 expression was associated with the malignant progression of HNSCC and indicated poor prognosis. The results of the in vitro and in vivo experiments verified that YOD1 depletion significantly promoted growth, invasion, and epithelial-mesenchymal transition in HNSCC. Mechanistically, YOD1 inhibited the activation of the ERK/β-catenin pathway by suppressing the ubiquitination and degradation of TRIM33, leading to the constriction of HNSCC progression. Overall, our findings reveal the molecular mechanism underlying the role of YOD1 in tumor progression and provide a novel potential therapeutic target for HNSCC treatment.

## Introduction

Head and neck cancer is the sixth most common type of cancer worldwide [1, 2] and is a heterogeneous cluster of cancers originating in the mucous membranes of the oral cavity, pharynx, and larynx [3]. More than three-quarters of head and neck squamous cell carcinoma (HNSCC) cases are due to tobacco and alcohol consumption [4, 5], and human papillomavirus has arisen as a new hazardous element for cancers [6]. Approximately 60% of HNSCC patients are diagnosed with advanced-stage disease, and >65% experience recurrence, develop metastases, or both. The five-year overall survival rate of patients with HNSCC remains under 50% although multimodal therapy, including combined surgical resection, chemo-radiotherapy, and immunotherapy, has improved the survival rate of patients [7, 8]. Survival outcomes remain poor for the great majority of patients, emphasizing the exigence to identify novel potential therapeutic targets which may extend lifetimes while improving quality of life [9].

The ubiquitin-proteasome system (UPS) controls proteolysis and plays a crucial role in adjusting intracellular protein balance. Ubiquitination is related to protein metabolism, apoptosis, cell cycle progression, differentiation, and metastasis [10]. Similar to other post-translational modifications (PTMs), ubiquitination is a reversible reaction where the removal of the modification is mediated by deubiquitinating enzymes (DUBs) which shear ubiquitin from the substrate protein, alter the peptidase, and compile the ubiquitin chain [11]. In previous studies, maintenance of the stem-cell-like properties of carcinoma can be achieved by the use of UCH-L3 to stabilize aromatic receptors [12]. DUB-2 can inhibit cell apoptosis [13]. USP15 stabilizes MDM2 to mediate cancer cell survival and inhibits antitumor T-cell responses [14]. Therefore, DUBs are very critical for the occurrence and development of tumors.

Here, we evaluated the levels of 95 DUBs by using bioinformatics analysis and found that YOD1 expression was dramatically diminished in HNSCC, and the low level of YOD1 correlated positively with lymph node metastasis and poor prognosis of HNSCC patients in our cohort. In addition, we verified that overexpression of YOD1 could restrain the proliferation and invasion abilities of HNSCC cells by regulating the TRIM33-mediated ERK/β-catenin pathway. Given its importance, YOD1 acts as a key DUB to suppress HNSCC progression.

## Materials and methods

### Bioinformatics analysis

We analyzed 95 potential DUBs using the public dataset GSE37991 concerning the expression profiles in tumor and non-tumor pair-wise samples from 40 male HNSCC patients. The online software GEPIA (gepia.cancer-pku.cn) was provided to analyze TCGA (The Cancer Genome Atlas) database. Survival analysis was performed using the Kaplan-Meier Plotter and GEO2R was used to identify gene expression values from GSE37991.

### Antibodies and reagents

The following antibodies were used for western blotting (WB), immunoblotting (IB), immunoprecipitation (IP), immunohistochemistry (IHC), and immunofluorescence (IF) assay in this study. From Proteintech (Rosemont, IL, USA): YOD1, 25370-1-AP (WB, 1:1000; IP, 1:200; IHC, 1:200); E-cadherin, 60335-1-Ig (WB, 1:1000; IHC, 1:200); Phospho-ERK1/2, 28733-1-AP (WB, 1:1000); and ZEB1, 21544-1-AP (WB, 1:1000). From Cell Signaling Technology: Erk1/2, #4695 (WB, 1:1000); Ubiquitin, #3936 (WB, 1:1000); Mouse IgG1 isotype control, #5414 (WB, 1:5000); Snail, #3879 (WB, 1:1000); Flag, #14793 (WB, 1:1000); Vimentin, #5741 (WB, 1:1000); Phospho-β-Catenin, #4176 (WB, 1:1000); β-Catenin, #8480 (WB, 1:1000); and Ki67, #9449 (IHC, 1:1000). From Abcam (Cambridge, UK): Twist, ab50887 (WB, 1:1000); N-cadherin, ab98952 (WB, 1:1000); From Santa Cruz (Dallas, Texas, USA): GAPDH, sc-365062 (WB, 1:5000) and TRIM33, sc-101179 (WB, 1:1000; IHC,1:200). MG132, CHX were bought from Selleck (Shanghai, China); 4-Nitroquinoline 1-oxide (4-NQO) was bought from Sigma-Aldrich (St. Louis, MO, USA).

### HNSCC specimens and immunohistochemical analysis

We obtained forty-one pairs of oral, laryngeal, or hypopharyngeal cancers and adjacent tissue samples from patients who had been operated on. All experiments performed on tissue samples were approved by the ethical committee of the Tianjin Medical University Cancer Institute and Hospital. and informed consent was obtained from the patients. HNSCC tissues were fixed with formalin, embedded in paraffin, dewaxed, hydrated, incubated with 0.3% hydrogen peroxide and goat serum, and then incubated with primary antibody at 4 °C overnight. Finally, samples were stained with DAB. ImageScope software (Leica Biosystems, Nussloch, Germany) was used to visualize and analyze the specimens. The IHC values were determined by the staining intensity as well as the proportion of positive tumor cells. The intensity of staining was scored as follows: 0 for negative, 1 for light staining (weak positive), 2 for moderate staining (positive), 3 for intense staining (strong positive). Additionally, the staining was graded according to the percentage of positive tumor cells: 0, no positive tumor cells; 1 to 4 means ≤ 25%, 26%-50%, 51%-75%, > 75% positive tumor cells, respectively. The final value for each case was obtained by multiplying the scores of staining intensity and proportion of positive tumor cells.

### Cell culture

SCC15- and SCC25-HNSCC cell lines were obtained from the American Type Culture Collection (ATCC, Manassas, VA, USA). To avoid cross-pollution and misrecognition of cell lines, DNA STR analysis was carried out. DMEM/F12 supplemented with 10% FBS and 1% penicillin/streptomycin was used to culture cells at 37 °C in a humid environment of 5% CO_2_. All cells were checked for mycoplasma contamination prior to the experiments.

### Transfection and transduction

Wild-type (WT) YOD1 and C120S mutant were cloned into a pLVX-IRES-puro vector. The shRNA sequences targeting YOD1 and TRIM33 were 5′-GGAGCAATAGAGATATCGA-3′ (shYOD1-2), 5′-GATGCAGGATATACCAAAA-3′ (shYOD1-3), 5′-GCCAGATATTCCACCCATA-3′ (shTRIM33-2), 5′-CGAAGCACATCAAAGAGTA-3′ (shTRIM33-3), which were cloned into the pSIH1-H1-puro vector. Lentivirus for inducible expression of YOD1 (Tet-On) was purchased from OBIO Technology (Shanghai, China). YOD1- and TRIM33- targeting small interfering RNAs (siRNAs), along with negative controls, were ordered from RIBOBIO (Guangzhou, China). The siRNA sequences used were listed in Supplementary Table S1. Transfection was performed using Lipofectamine 2000 according to the standard protocols, and transduction was conducted as previously described [15].

### Western blotting

Cells were cleaved on ice with a lysate containing protease and phosphatase inhibitors for 30 min and were then centrifuged at 14,000 RPM for 20 min (4 °C) to collect the supernatant. Concentrations were determined using a BCA protein assay kit (Thermo Fisher Scientific, Waltham, MA, USA). Gel electrophoresis was performed after denaturation. After conversion, 5% skim milk was added for 1 h and samples were incubated at 4 °C for 14 h with primary antibody.

### Quantitative real-time PCR (qPCR)

Total RNA was extracted from the cells using TRIzol reagent (Invitrogen, Waltham, MA, USA) according to standard instructions and then reverse-transcribed to cDNA using PrimeScript™ RT Master Mix (TaKaRa, Shiga, Japan). Expression analysis was performed on a Q5 real-time PCR system (Applied Biosystems, Foster City, CA, USA) using SYBR Premix Ex Taq II (TaKaRa). GAPDH served as a loading control to normalize the expression levels of target genes. The relative abundance of the genes was determined using the 2−ΔΔCt method. The primers used for GAPDH and the target genes were listed in Supplementary Table S2.

### MTT assay

For cell growth detection, the cells were seeded in 96-well plates and stained at the specific time points using MTT dye for 5 h at 37 °C. After staining, the culture medium was removed and 150 µL DMSO was added. The OD at 490 nm was tested using a microplate reader (Model 680; Bio-Rad Laboratories Ltd., Hercules, California, USA).

### Clonogenic assay

Cells were seeded in a six-well plate (1000 cells per well) and cultured for 2 weeks. Afterward, cells were washed twice with PBS and fixed with methanol. Colonies were stained with 0.1% crystal violet and counted (>50 cells).

### Ethynyl-20-deoxyuridine (EdU) assay

EdU Imaging Kits (Cy3) (APExBIO, Houston, Texas, USA) were used for assessing cell proliferation. HNSCC cells were seeded in a 24-well plate (2 × 10^4^ cells per well) and incubated overnight. After re-incubation with the EdU reagent for 2 h, and after fixing and staining, nucleic acid was stained with Hoechst 33342. Images were acquired using a fluorescence microscope (Leica DMI6000B).

### Wound-healing assay

HNSCC cell lines, SCC15 and SCC25, were seeded into six-well plates. When the cell density was close to 100%, wells were scratched with 10 µL pipette tips. The cells were then washed and cultured in a fresh medium without FBS for another 24 h. Plates were imaged at 0 h and 24 h.

### Transwell assay

For in vitro invasion or migration assays, HNSCC cells (SCC15, 1 × 10^5^ cells; SCC25, 1 × 10^5^ cells) were seeded into a transwell chamber pre-coated with Matrigel or not. To the bottom chambers, 600 µl medium containing 20% fetal bovine serum was added. The transwell cells were incubated at 37 °C for 36 h. After, cells were immobilized with methanol and stained with 0.1% crystal violet. An inverted microscope was used to image and count the cells.

### Immunofluorescence staining

HNSCC cells were seeded onto 18 mm cover glass and incubated overnight at 37 °C. After methanol immobilization, the cells were permeabilized with 0.2% Triton X-100. Cells were then sealed with 2% BSA and incubated with primary antibodies at 4 °C for 14 h. Cells were then incubated with anti-rabbit IgG conjugated to Alexa Fluor 488 or anti-mouse IgG conjugated to Alexa Fluor 594 (Cell Signaling Technology) for 60 min at room temperature, and the nuclei were stained with 4,6-diamidino-2- phenylindole (DAPI; Thermo Fisher Scientific) for another 10 min. All images were captured using an Axio Imager Z2 microscope (Zeiss, Oberkochen, Germany).

### Mass spectrometry

SCC15 cells expressing Flag-YOD1 or negative control were lysed, then the cell lysates were immunoprecipitated with anti-FLAG M2 affinity gel (A2220, Sigma-Aldrich). After gel electrophoresis, enzymatic hydrolysis in gum and desalting was performed, and the digested sample was injected into a Nano-LC system (EASY-nLC 1200, Thermo Fisher Scientific). The HPLC elute was electrosprayed directly into an Orbitrap Q-Exactive Plus mass spectrometer (Thermo Fisher Scientific). The source was operated at 2.4 kV. The mass spectrometric analysis was carried out in a data-dependent mode. LC-MS/MS data were searched using Thermo Proteome Discoverer 1.4 with an overall false discovery rate (FDR) for peptides of less than 1%.

### Co-immunoprecipitation assay

For endogenous immunoprecipitation, the HNSCC cell lysates were incubated overnight with 2 μg anti-YOD1 antibodies or IgG and then conjugated with 30 ml 50% Protein A/G PLUS-Agarose (sc-2003, Santa Cruz) for an additional 2 h incubation. After washing the beads three times, the immunoprecipitated proteins were then analyzed by immunoblotting.

### In vivo ubiquitination assay

To investigate endogenous TRIM33 ubiquitination, cells were incubated with MG132 (10 μM) for 12 h and lysed with RIPA buffer. Anti-TRIM33 antibody was applied to immunoprecipitating proteins from the cell lysate and separate ubiquitinated TRIM33. The endogenous ubiquitin chains on TRIM33 were identified by immunoblotting with an antibody against ubiquitin.

### Xenograft models

All experimental protocols were in accordance with the requirements of the Cancer Institute of Tianjin Medical University and the Animal Care and Use Committee.

For the 4-NQO-induced HNSCC animal model, six-week-old mice (C57BL/6 mice) were fed with water containing 4-NQO (50 µg/mL) for 16 weeks and then fed with normal water for another 12 weeks. Tongues were removed at week 28 for further study.

For the in vivo growth assay, BALB/c nude male mice (4 weeks old) were double-blindly, randomly divided into two groups (seven per group), and housed in a specific pathologic-free environment. SCC15 cells stably expressing YOD1 or negative control were injected into the instep of the mice using 1 × 10^5^ cells per mouse. The measurement of tumor volume was conducted every 3 days, and the groin and intermuscular lymph nodes were palpated. 20 days later, the mice were euthanized. The tumor tissues were separated, measured, fixed, and embedded in paraffin for IHC detection. The lymph nodes were stained with hematoxylin and eosin.

### Statistical analysis

Data analysis was conducted using the GraphPad Prism software (version 8.0; CA, USA). Data are expressed as mean ± standard deviation. Comparison between the two groups was performed by Student’s *t* test. Proliferation curves were analyzed using two-way ANOVA. Survival curves were calculated using Kaplan–Meier analysis, and the correlation between YOD1 expression and clinicopathological features of HNSCC patients was analyzed using Spearman’s rank correlation analysis, which was followed by a linear correlation analysis when the Spearman correlation was significant. Statistical significance was set at *P* < 0.05.

## Results

### Low expression of YOD1 indicates poor prognosis of HNSCC patients

**Fig. 1:**
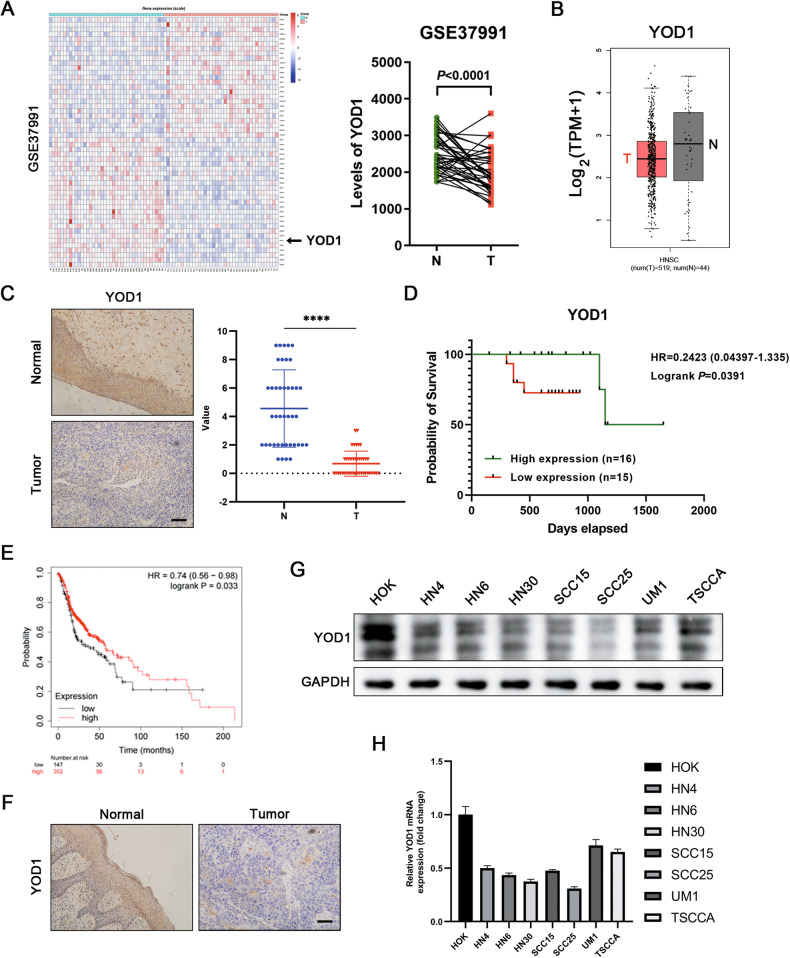
YOD1 reduction predicts poor overall survival in HNSCC. **A** The cluster of heat map identified differentially expressed DUBs in HNSCC by analyzing GEO dataset GSE3799. YOD1 was overexpressed in adjacent normal tissues compared to HNSCC tissues. N normal tissues. T tumor tissues. Data, mean ± SD. **B** The level of YOD1 in normal tissues and HNSCC tissues through the bioinformatics analysis of TCGA database. Data, mean ± SD. T tumor tissues. N normal tissues. TPM transcripts per million. **C** The expression of YOD1 in adjacent normal tissues and HNSCC specimens was measured using IHC assay. Scale bar, 50 μm. N normal tissues. T tumor tissues. Data, mean ± SD, ****P < 0.0001. **D** Kaplan–Meier survival curve showed that reduced expression of YOD1 indicated unfavorable prognosis in HNSCC. **E** The correlation between the expression of YOD1 and overall survival in HNSCC was assessed via Kaplan–Meier plotter database. HR, hazard rate. **F** A 4-NQO-induced murine HNSCC model was established and tongue specimens of mice were collected at Week 28. The results of IHC showed that YOD1 expression in HNSCC was lower than that in normal tissues. Scale bar, 50 μm. **G** Western blot assay was performed to measure the protein level of YOD1 in HOK cell line and a panel of HNSCC cell lines. **H** Expression of YOD1 mRNA in HOK cell line and HNSCC cell lines. Data, mean ± SD.

The expression profiles of 95 DUBs from the GSE37991 Gene Expression Omnibus (GEO) datasets were analyzed to identify statistically significant DUBs expressed in HNSCC tissues (Fig. 1A). The GEO data analysis showed that in patients suffering with HNSCC, YOD1 expression was lower in cancer tissues compared with adjacent tissues (Fig. 1A). Moreover, TCGA database analysis showed that the level of YOD1 was reduced in HNSCC relative to normal tissues (Fig. 1B). Then we studied 41 pairs of clinical specimens from patients diagnosed with HNSCC. From the results of IHC, the expression level of YOD1 in cancer tissues was clearly lower than that in healthy tissues. The location of YOD1 was cytoplasmic (Fig. 1C). Subsequent analysis showed that YOD1 was markedly associated with T stage and lymph node metastasis (Table 1). Furthermore, Kaplan-Meier analysis showed that HNSCC patients with lower YOD1 expression showed worse outcomes (Fig. 1D), which was reconfirmed by the Kaplan-Meier Plotter analysis of HNSCC (Fig.1E). We then set up a murine model induced by 4-NQO and obtained tongue samples from the seven mice at week 28. The IHC results confirmed that YOD1 protein expression was much lower in cancer tissues than in normal tissues (Fig. 1F), suggesting that YOD1 may participate in HNSCC tumor progression. Thus, YOD1 may be a potential tumor-suppressive gene in HNSCC.

**Table 1:** YOD1 expression and clinicopathological features of HNSCC.

Clinicopathological features	YOD1 expression		Total	*P* value
	Low	High		
Overall	22 (53.7%)	19 (46.3%)	41	
Gender				
Male	22 (56.4%)	17 (43.6%)	39	0.209
Female	0 (0%)	2 (100.0%)	2	
Age				
≤60	14 (58.3%)	10 (41.7%)	24	0.476
>60	8 (47.1%)	9 (52.9%)	17	
Clinical stage				
I–II	18 (54.5%)	15 (45.5%)	33	0.817
III–IV	4 (50.0%)	4 (50.0%)	8	
T stage				
T1-T2	13 (40.6%)	19 (59.4%)	32	0.002^*^
T3-T4	9 (100.0%)	0 (0%)	9	
LN metastasis				
No	11 (36.7%)	19 (63.3%)	30	<0.001^*^
Yes	11 (100.0%)	0 (0%)	11	
Recurrence				
No	15 (45.5%)	18 (54.5%)	33	0.050
Yes	7 (87.5%)	1 (12.5%)	8	

The result was analyzed by the Pearson *χ*^2^ test. *P* values with significance were shown as an asterisk. ^*^P < 0.05.

### YOD1 inhibits HNSCC cell proliferation in vitro

**Fig. 2:**
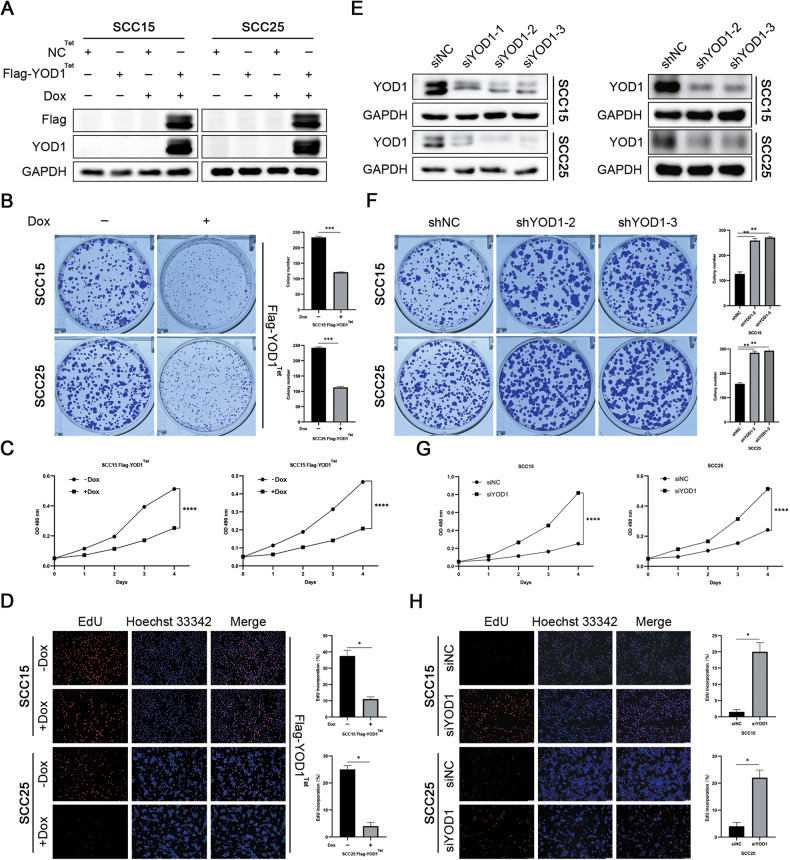
YOD1 inhibits the proliferation of HNSCC cells in vitro. **A**, **E** The protein expressions of YOD1 were measured in the SCC15 and SCC25 cells expressing Dox-induced YOD1 (**A**), shRNAs targeting YOD1, or transfected with siYOD1 (**E**). **B**, **F** Clonogenicity assays for SCC15 and SCC25 cells with YOD1 overexpression (**B**) or knockdown (**F**). **C**, **G** MTT assays for SCC15 and SCC25 cells with YOD1 overexpression (**C**) or knockdown (**G**). **D**, **H** EdU assays for SCC15 and SCC25 cells with YOD1 overexpression (**D**) or knockdown (**H**). Scale bar, 100 μm. Data in this figure, mean ± SD, *P < 0.05, **P < 0.01, ***P < 0.001, and ****P < 0.0001. Dox doxycycline.

We tested the protein expression of YOD1 in a normal human oral keratinocyte (HOK) cell line and a panel of HNSCC cell lines. As shown by WB (Fig. 1G) and qPCR (Fig. 1H), the expression of YOD1 in HOK cells was significantly higher than that in HNSCC cells. Compared to other cell lines, SCC15 and SCC25 cell lines showed lower expression of YOD1, and were therefore selected for further experiments. In order to exclude the impact of plasmid toxicity on cell growth, we established cell lines expressing doxycycline-induced Flag-YOD1 (Fig. 2A). Compared with HNSCC cells treated with Dox, the number of cell colonies in the control cell lines was significantly larger (Fig. 2B). In addition, the growth rate of cells exposed to Dox was lower than that of cells in the control group (Fig. 2C). In the EdU assay, Dox-induced YOD1 significantly inhibited cell proliferation (Fig. 2D). In addition to knocking down YOD1 using a mixture of three siRNAs, we also generated stable cell lines expressing shRNAs targeting YOD1 (Fig. 2E). The results (Fig. 2F–H) were quite the opposite of the previous results (Fig. 2B–D). Collectively, these data show that YOD1 dramatically inhibits cell proliferation and colony formation in HNSCC.

### YOD1 inhibits migration and invasion of HNSCC cells in vitro

**Fig. 3:**
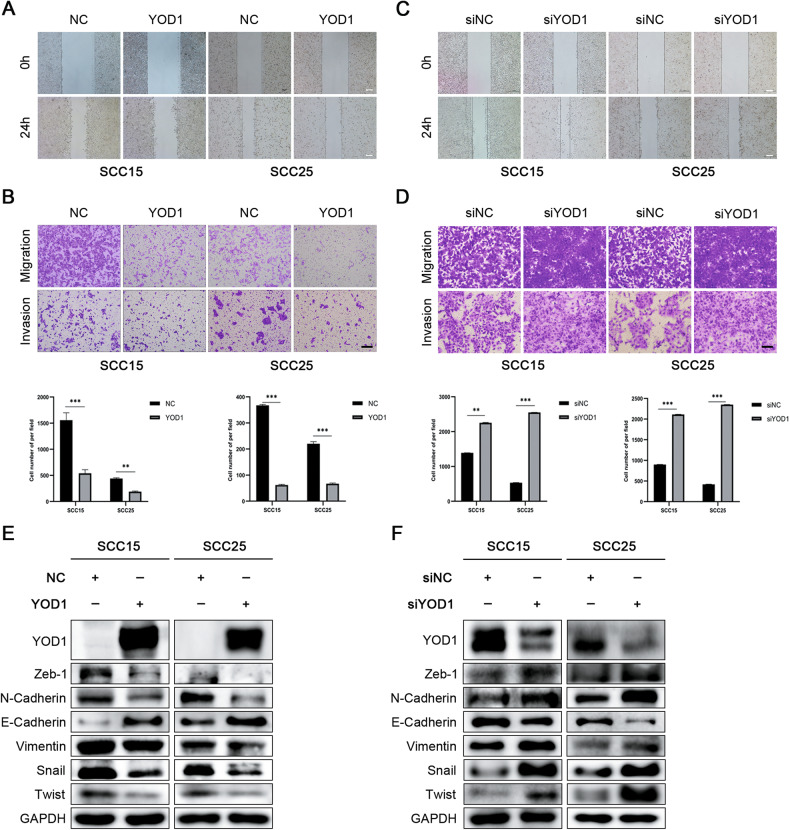
YOD1 inhibits the migration and invasion of HNSCC cells in vitro. **A**, **C** Wound-healing assays for SCC15 and SCC25 cells with YOD1 overexpression (**A**) or downregulation (**C**). Scale bar, 200 μm. **B**, **D** Transwell assays for SCC15 and SCC25 cells with YOD1 overexpression (**B**) or downregulation (**D**). Scale bar, 100 μm. **E**, **F** Immunoblotting of EMT markers and EMT-TFs for SCC15 and SCC25 cells with YOD1 overexpression (**E**) or downregulation (**F**). Data in this figure, mean ± SD, *P < 0.05, **P < 0.01, and ***P < 0.001.

Next, the effect of YOD1 on the migration and invasion of HNSCC cells was explored through several experiments. According to the wound-healing and transwell assays shown in Fig. 3A, B, cell migration and invasion were strikingly blocked after the upregulation of YOD1. However, opposite outcomes were observed in the two cell lines after transfection, as YOD1 deficiency obviously accelerated migration and invasion in SCC15 and SCC25 cells (Fig. 3C, D). Furthermore, overexpression of YOD1 clearly increased the expression of epithelial marker E-cadherin and reduced the expression of mesenchymal markers N-cadherin and vimentin in SCC15 and SCC25 cells (Fig. 3E). We detected the expression of epithelial-to-mesenchymal transition transcription factors (EMT-TFs) in SCC15 and SCC25 cells. YOD1 overexpression dramatically lessened the protein expression levels of Snail, Twist, and ZEB1 (Fig. 3E). In turn, the knockdown of YOD1 rescued these changes (Fig. 3F). All results indicate that YOD1 inhibits the migration and invasion of HNSCC cells.

### YOD1 stabilizes the E3 ligase TRIM33 and inhibits the ERK/β-catenin signaling pathway

**Fig. 4:**
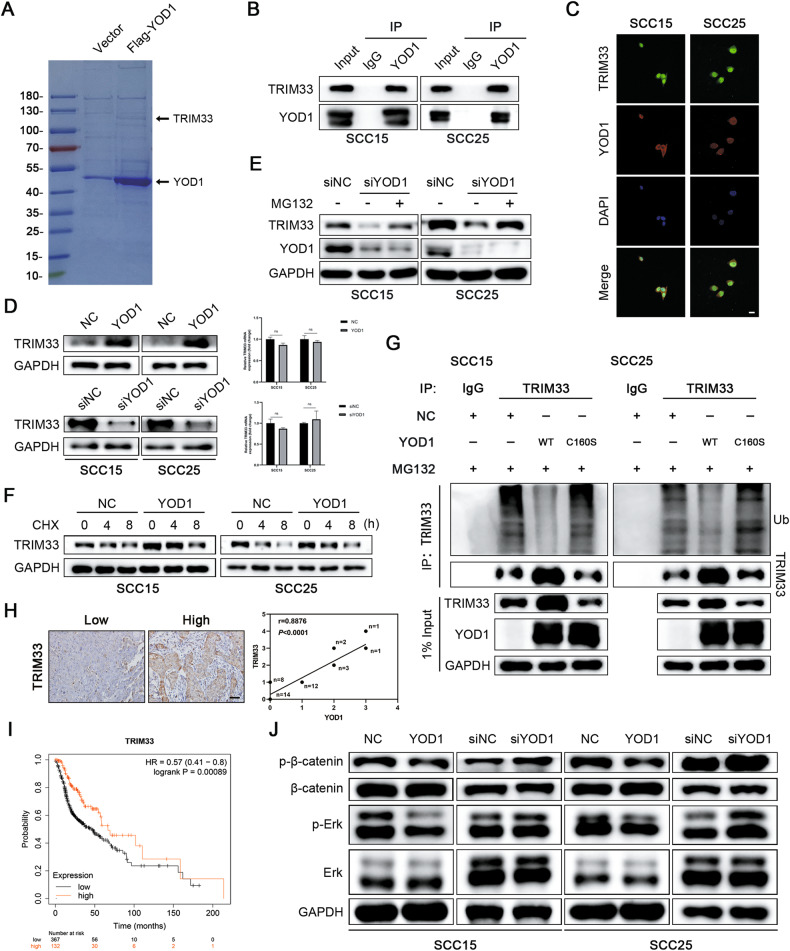
YOD1 stabilizes TRIM33 and inhibits the ERK/β-catenin signaling pathway. **A** The protein was separated by SDS-PAGE and analyzed by HPLC-MS. **B** Endogenous YOD1 protein were immunoprecipitated with anti-Flag antibody or IgG, and then analyzed by immunoblotting assay. **C** The representative images of immunofluorescence staining of YOD1 and TRIM33 in HNSCC cells. Scale bar, 20 μm. **D** The expressions of TRIM33 protein and mRNA were detected in HNSCC cells with YOD1 overexpression or YOD1 knockdown. Data, mean ± SD, ns, no significance. **E** The immunoblotting assay showed that MG132 mitigated the inhibition of siYOD1 on TRIM33. **F** SCC15 and SCC25 cells expressing YOD1 were exposed to 0.05 mg/ml CHX for 0, 4, 8 h. The TRIM33 protein expression was analyzed by immunoblotting assay. **G** The poly-ubiquitination level of endogenous TRIM33 in HNSCC cells stably expressing YOD1, catalytic inactive mutant of YOD1 (C160S), and negative control was assessed by in vivo ubiquitination assay. 1% input of cell lysates was used to assess the expression of TRIM33 and YOD1. **H** IHC staining showed that there was a significantly positive correlation between YOD1 and TRIM33 in HNSCC. Scale bar, 50 μm. **I** Kaplan–Meier survival curve showed that a low level of TRIM33 indicated poor prognosis in HNSCC. **J** The abundance of β-catenin, p-β-catenin, ERK, and p-ERK was measured in SCC15 and SCC25 cells expressing YOD1 transfected with siYOD1.

To obtain a more in-depth understanding of the mechanisms underlying YOD1 function, an IP-MS assay was performed. The results reveal that YOD1 inhibited the expression of EMT-TFs, and generally inhibited protein degradation and improved protein stability; other E3 ligases involved in the adjustment of EMT-TFs expression have previously been identified [16–18]. Therefore, we hypothesized that YOD1 may regulate some E3 ligases to promote protein degradation of EMT-TFs. According to the results of liquid chromatography-mass spectrometry analysis (Fig. 4A), we found that the E3 ligase TRIM33 may bind to YOD1. Immunoprecipitation and fluorescence co-localization assays were used to verify that YOD1 was directly bound to TRIM33 (Fig. 4B, C). The WB results confirmed that the overexpression of YOD1 clearly increased TRIM33 instead of other E3 ligase candidates (Fig. 4D and Supplementary Fig. 1), but the enzyme-deficient mutant (YOD1 C160S) had little effect on the protein level of TRIM33 (Supplementary Fig. 1). Inversely, loss of YOD1 decreased TRIM33 obviously (Fig. 4D). However, there was no obvious alteration in the mRNA levels of TRIM33 (Fig. 4D). Furthermore, after treatment with MG132, the degradation of TRIM33 was significantly inhibited (Fig. 4E). Additionally, under the effect of CHX, experimental results showed that YOD1 overexpression extended the half-life of TRIM33 and retarded its degradation (Fig. 4F). As shown in Fig. 4G, the ubiquitination level of TRIM33 was significantly reduced by wild-type YOD1 instead of C160S mutant, indicating that deubiquitinating enzyme activity was required for the effect of YOD1 on TRIM33 (Fig. 4G). Meanwhile, IHC staining showed that YOD1 had a positive correlation with TRIM33 in HNSCC samples (Fig. 4H). Although no obvious difference in TRIM33 mRNA level was observed between normal tissues and HNSCC tissues through TCGA database analysis (Supplementary Fig. 2), Kaplan-Meier Plotter analysis demonstrated that the low expression of TRIM33 was related to poor prognosis of HNSCC (Fig. 4I). Previous studies have shown that ERK and β-catenin signaling pathways play crucial roles in the malignant progression of multiple cancers including HNSCC. Therefore, we explored whether YOD1 had an effect on these pathways. Using the western blot, we found that YOD1 inhibited the activation of ERK and β-catenin signaling pathways in HNSCC cell lines (Fig. 4J and Supplementary Fig. 3).

### YOD1 acts as a cancer suppressor through TRIM33

**Fig. 5:**
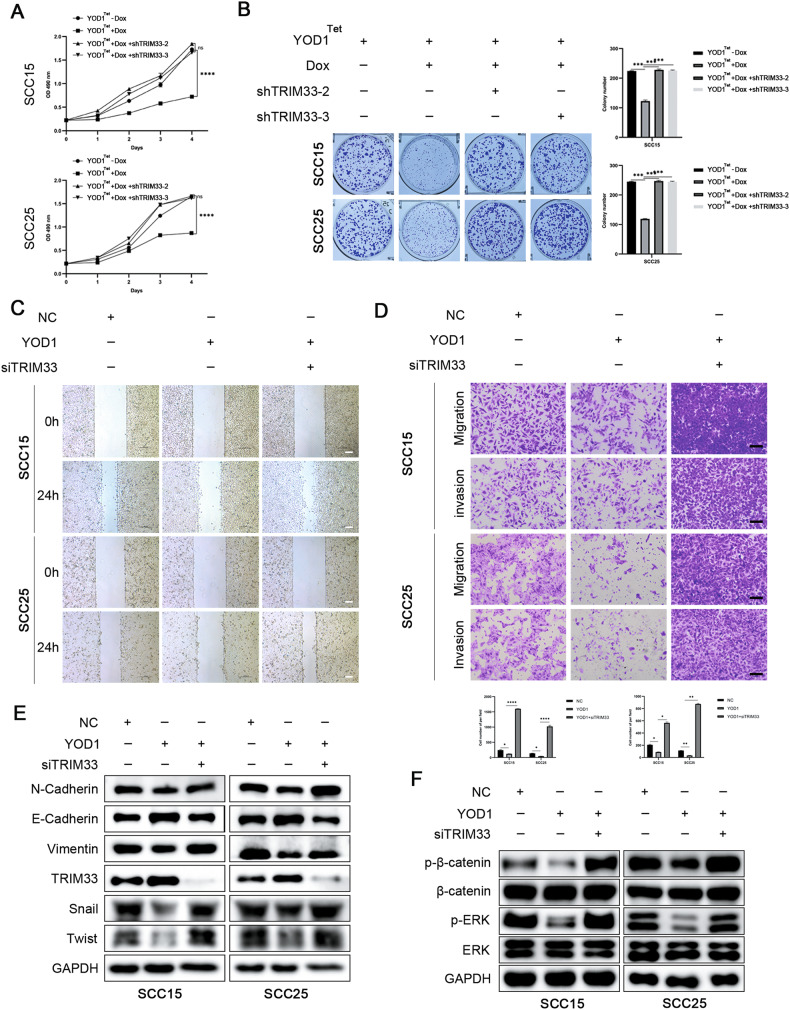
YOD1 inhibits malignant progression of HNSCC by regulating TRIM33. **A** MTT assay was used to detect the effect of shTRIM33 on SCC15 and SCC25 cells after Dox-induced overexpression of YOD1. **B** Clonogenicity assay was used to detect the effect of shTRIM33 on SCC15 and SCC25 cells after Dox-induced overexpression of YOD1. **C** Wound-healing assay was performed in TRIM33-depleted SCC15 and SCC25 cells expressing YOD1. Scale bar, 200 μm. **D** Transwell assay was conducted to detect the capacities of migration and invasion of TRIM33-silenced SCC15 and SCC25 cells stably expressing YOD1. Scale bar, 100 μm. **E** Immunoblotting was used to assess the levels of EMT markers and EMT-TFs in SCC15 and SCC25 cells as indicated. **F** Immunoblotting was used to detect β-catenin, p-β-catenin, ERK, and p-ERK in indicated SCC15 and SCC25 cells. Data in this figure, mean ± SD, *P < 0.05, **P < 0.01, ***P < 0.001, and ****P < 0.0001. Dox doxycycline.

For further confirming that YOD1 inhibited malignancy of HNSCC cells through regulating TRIM33, TRIM33 knockdown was performed in HNSCC cells stably expressing YOD1. Using MTT and clonogenic assays, we observed that the inhibitory effect of YOD1 on HNSCC cell proliferation was partially restored by TRIM33 knockdown (Fig. 5A, B). Similarly, TRIM33 knockdown also restored the migration and invasion ability of YOD1 on HNSCC cells (Fig. 5C, D). The trends in EMT markers and EMT-TFs also supported these changes (Fig. 5E). The deletion of TRIM33 also restored the inhibitory effect of YOD1 on the activation of the above pathways to a certain extent (Fig. 5F).

### Overexpression of YOD1 attenuates tumor growth and lymph node metastasis of HNSCC cells in vivo

**Fig. 6:**
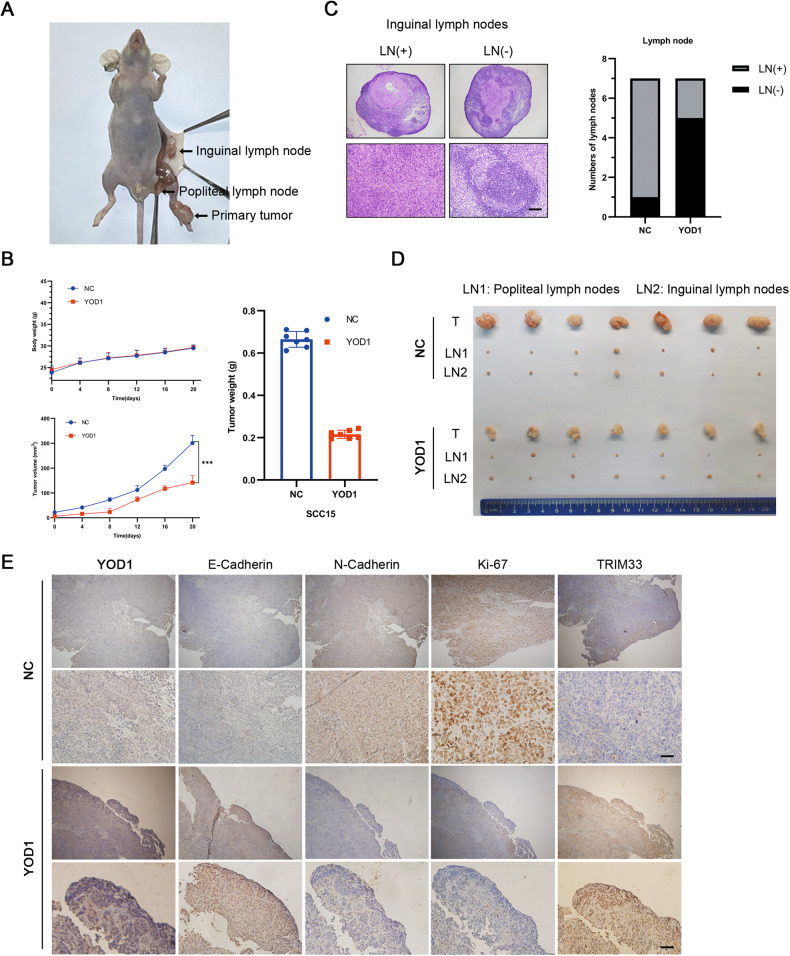
Overexpression of YOD1 attenuates tumor growth and Lymph node metastasis of HNSCC cells in vivo. **A** Representative images of xenografts and lymph node metastases in mice. **B** The weight of body and xenograft, and tumor volume were measured in YOD1 overexpression group and negative control (*n* = 7). **C**, **D** Representative images of lymph node metastasis in indicated groups. Scale bar, 100 μm. **E** The levels of YOD1, E-Cadherin, N-Cadherin, Ki67, and TRIM33 in indicated groups were measured by using IHC staining. Scale bar in **C**, **E**, 50 μm. Data in this figure, mean ± SD, ***P < 0.001.

Finally, a xenograft model was used to examine the effect of YOD1 in HNSCC in vivo. The results showed that the weight of xenografts from YOD1 overexpressing SCC15 cells was notably lower than that come from control cells (Fig. 6B), indicating the tumor-suppressive effect of YOD1 on HNSCC tumor growth. Additionally, the number of lymph node metastases in the YOD1 overexpression group was significantly lower than that in the control group (Fig. 6C, D). IHC staining showed that the expression of E-cadherin and TRIM33 was enhanced while the levels of N-cadherin and Ki67 decreased in the control group (Fig. 6E), indicating that YOD1 inhibited EMT and the proliferation of HNSCC cells. Collectively, these results illustrate that YOD1 acts as a tumor suppressor in vivo.

## Discussion

Proteases are involved in multiple biological and pathological procedures [19] and are important possible targets for the development of diverse antineoplastic drugs [20]. DUBs, a large class of proteases, rely on the UPS and control the abundance of a variety of proteins. Multiple diseases, including cancer, have been proposed to be associated with the aberrant biological activity of DUB. Recent research has demonstrated that some DUBs can be classified as oncogenes or suppressor genes [21]. For instance, BAP1’s role in the deubiquitination of KLF5 encourages the progression of breast carcinoma [22]. For colorectal carcinoma prevention, potential therapeutic strategies have been identified by controlling FEW7 stability with FAF [23]. In addition, USP21 encourages malignant HIF-1-driven metabolic reprogramming and may be a target for pancreatic carcinoma treatment [24]. These DUBs play pro-cancer or anti-cancer roles in different cancers, and it is worth noting that the same DUBs may have opposite effects in different cancers. A study published in Cell Reports revealed the important antitumor function of the deubiquitinating enzyme OTUB2 in oral and esophageal squamous cell carcinomas by promoting phosphorylation and dimer formation of the transcription factor STAT1 [25]. However, in a study of breast cancer, OTUB2 promoted metastasis through hippocampal-independent activation of YAP and TAZ [26]. This difference may be caused by the different tumor contexts and target proteins.

It has previously been reported that YOD1 is closely associated with multiple tumors, including liver cancer, gallbladder cancer, pancreatic adenocarcinoma, acute promyelocytic leukemia, osteosarcoma, and ovarian cancer [27–32]. For example, YOD1 regulates the Hippo pathway that promotes hepatocyte proliferation through the YOD1−ITCH−YAP/TAZ signaling axis [27]. Additionally, lncRNA FIRRE encourages the progression of gallbladder carcinoma through mediation of YOD1 expression when it functions as a miR-520a-3p sponge [28]. Moreover, aberrant expression of YOD1 is able to accelerate the progression of pancreatic carcinoma [29]. In acute promyelocytic leukemia (APL), YOD1 raises the PML/RARa oncoprotein’s stability; therefore, a selective YOD1 inhibitor named ubiquitin isopeptidase inhibitor I (G5) may offer the chance to treat APL which is resistant to treatment [30]. According to one study, MiR-4429 inhibits the growth of ovarian carcinoma by targeting YOD1 [32]. Our results demonstrated that in contrast to adjoining normal tissues, YOD1 was expressed at low levels in HNSCC tissues. The experimental outcomes show that high expression of YOD1 inhibits the malignant progression of HNSCC, and that low expression of YOD1 was related to poor prognosis. Further research emphasized this theory that YOD1 inhibited the progression of HNSCC. Therefore, YOD1 plays an antitumor effect in HNSCC.

TRIM33 belongs to the transcription intermediate factor 1 (TIF1) family of chromatin-binding proteins, a subfamily of the tripartitemotif (TRIM) family of E3 ligases [33]. Compared to other TIF1 family members, TRIM33 mainly plays a tumor suppressor role. Inhibition of the malignant progression of lung adenocarcinoma is achieved by TIFI-γ, which can regulate IL-6 transactivation by disrupting TAF15/tbp [34]. Reduced TIF1-γ also promotes the metastatic capacity of hepatocellular cancer by facilitating Smad2/3/4 complex formation, resulting in poor prognosis [35]. Research into clear cell renal cell carcinoma (ccRCC) showed that TRIM33 is upregulated by treatment with an inhibitor named miR-629, which notably suppresses TGFβ-induced Smad activation as well as facilitating metastasis of ccRCC [36]. In HNSCC, the function of YOD1, as well as the regulatory mechanism of its PTMs, has not been widely studied. This study revealed that YOD1 stabilizes TRIM33 to inhibit the ERK/β-catenin pathway, thereby acting as an antitumor gene in HNSCC.

EMT is an elementary biological process with a crucial role in embryogenesis. When EMT is reactivated during cancer development, illness progression, and a metastatic phenotype is promoted in benign carcinoma cells due to the development of traits such as migration and invasion. The activation of EMT-TFs, such as Snail, Twist, and Zeb1, gives cancer cells the ability to invade and migrate. However, EMT-TFs are highly labile proteins whose levels are controlled by the UPS. EMT-TFs are involved in maintaining the regulatory balance between DUB and E3 ligases; in turn, they can directly affect the expression of EMT-TFs, thus, influencing the metastatic phenotype of cancer. A study on breast cancer showed that TRIP12 suppresses EMT via ZEB1/2 [37]. Stabilization of Snail by USP26 can encourage ESCC metastasize to a certain extent [38]. Further, a variety of DUBs have been found to indirectly affect the expression of EMT-TFs by stabilizing E3 ligases, thereby further regulating the metastatic ability of tumors. For example, the antagonistic relationship between USP39 and TRIM26, as opposed to a competitive relationship, plays a critical role in ZEB1 stability [16]. In this research, we found that YOD1 indirectly affected the expression of EMT-TFs, such as Snail and Twist, through the E3 ligase TRIM33, thereby affecting HNSCC metastasis.

During tumor progression, cancer cells undergo characteristic changes in proliferation, survival, metabolism, migration, and differentiation, which implies changes in the cell signaling pathways controlling these properties in normal cells. Various cell signaling pathways are interconnected and constitute complex signaling pathways. Previous studies have clarified the detailed roles of the ERK [39] and β-catenin [40] signaling pathways in the micro-regulation of extracellular signaling. In this study, the experimental results showed that YOD1 modified TRIM33 through deubiquitination, maintaining the stability of TRIM33 and thus inhibiting the proliferation and metastasis of HNSCC cells by inhibiting the activation of ERK and β-catenin signaling pathways.

In summary, there is a positive relationship between the downregulation of YOD1 expression and poor prognosis in HNSCC patients. This strongly implies the clinical prognostic significance of YOD1 since it functions as an antitumor factor by stabilizing the E3 ligase TRIM33 in HNSCC.

## Supplementary information

Supplementary materials

Full uncut gels

## Data Availability

All data generated or analyzed during this study are included in this published article and supplementary information files.
