# Supplementary material for: Deubiquitinase YOD1 suppresses tumor progression by stabilizing E3 ligase TRIM33 in head and neck squamous cell carcinoma
Source: Cell Death Dis. 2023 Aug 12;14(8):517. doi: 10.1038/s41419-023-06035-0 (PMC10423255; doi:10.1038/s41419-023-06035-0)
Supplement: Supplementary file 1 — Supplementary materials [file 41419_2023_6035_MOESM1_ESM.docx]

**Supplementary Table S1. List of siRNAs sequence.**

| siRNA name | Sequence (5’-3’) |
| --- | --- |
| siYOD1-1 | GGGATACCATTCTGGAAGA |
| siYOD1-2 | GGAGCAATAGAGATATCGA |
| siYOD1-3 | GATGCAGGATATACCAAAA |
| siTRIM33-1 | GGAAGATGCTGGCTCAAGT |
| siTRIM33-2 | GCCAGATATTCCACCCATA |
| siTRIM33-3 | CGAAGCACATCAAAGAGTA |

**Supplementary Table S2. List of qPCR primers sequence.**

| Primer name | Sequence (5’-3’) |
| --- | --- |
| GAPDH | F: TGCACCACCAACTGCTTAGC |
|  | R: GGCATGGACTGTGGTCATGAG |
| YOD1 | F: AAACGTGGTGCTTCTAGTTACG |
|  | R: CTTCGACGACATAGTACACAC |
| TRIM33 | F: ATGTGGAGAGTGGCTATGTAAGA |
|  | R: GGGCGTTGACCAGATGCTC |


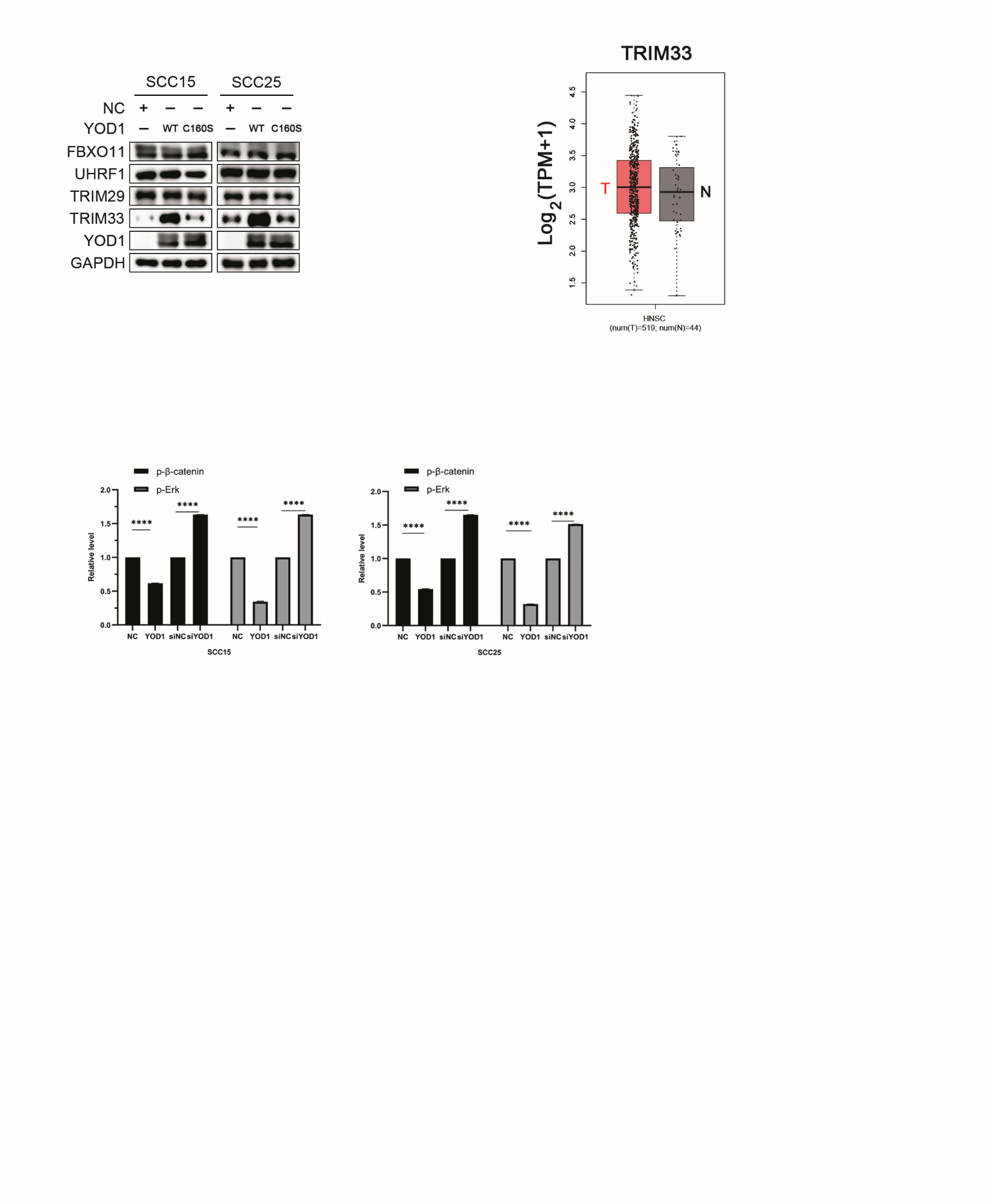


**Supplementary Fig. 1 YOD1 promotes the protein expression of TRIM33 instead of other E3 ligase candidates identified by Mass spectrometry.** The enzyme-dead mutant of YOD1 (C160S) failed to affect the expression of TRIM33. WT, wild-type.


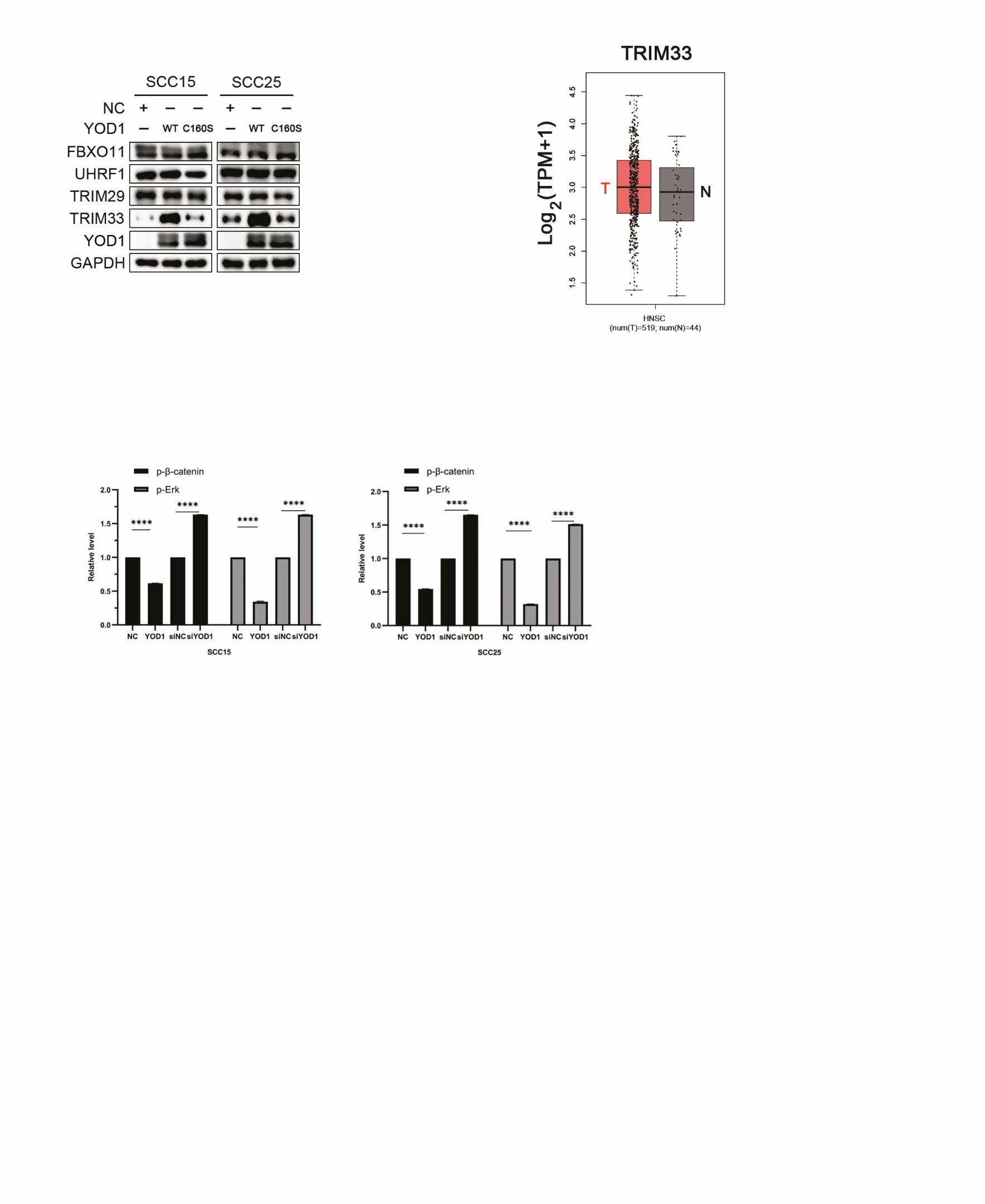


**Supplementary Fig. 2 The level of TRIM33 in normal tissues and HNSCC specimens based on the bioinformatics analysis of TCGA database.** Data, mean ± SD. T, tumor tissues. N, normal tissues. TPM, transcripts per million.


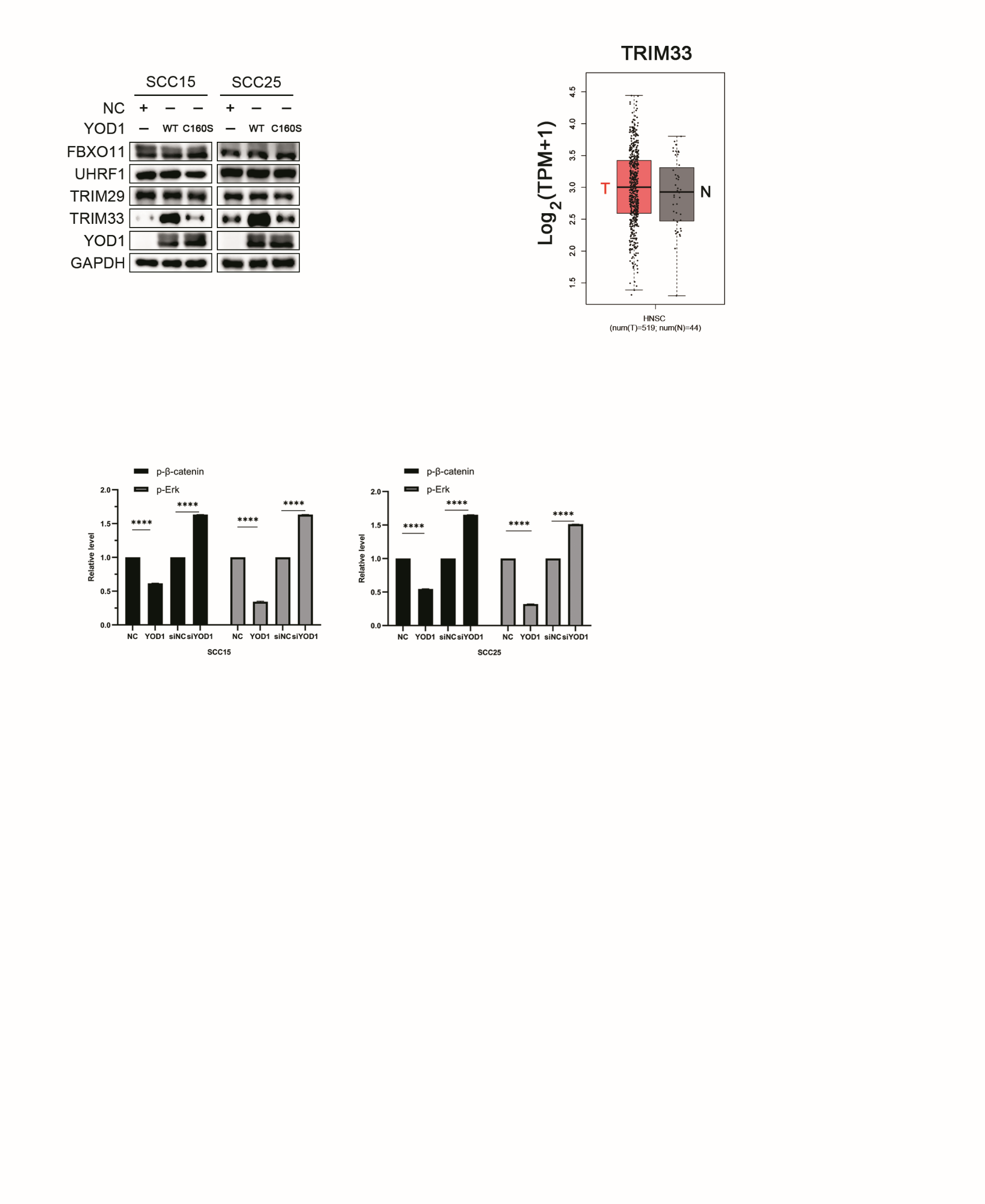


**Supplementary Fig. 3 The quantitative analysis of p-ERK and p-β-catenin detected in Figure 4J*.*** Data, mean ± SD, *****P*<0.0001.
