# Supplementary material for: Deubiquitinase YOD1 suppresses tumor progression by stabilizing E3 ligase TRIM33 in head and neck squamous cell carcinoma
Source: Cell Death Dis. 2023 Aug 12;14(8):517. doi: 10.1038/s41419-023-06035-0 (PMC10423255; doi:10.1038/s41419-023-06035-0)

Figure1-G

YOD1

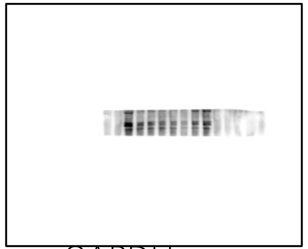

GAPDH

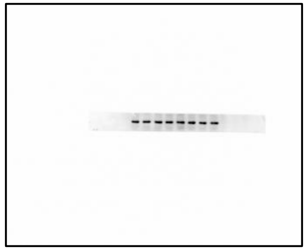

Figure2-A

GAPDH-SCC15

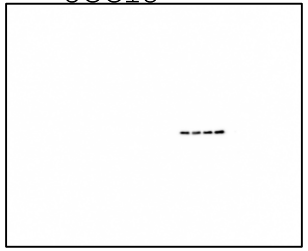

YOD1-SCC15

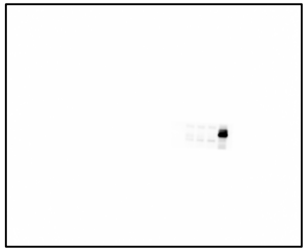

Flag-SCC15

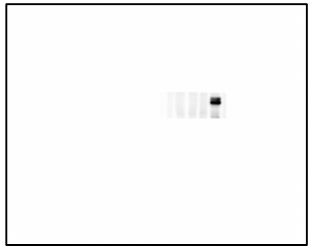

GAPDH-SCC25

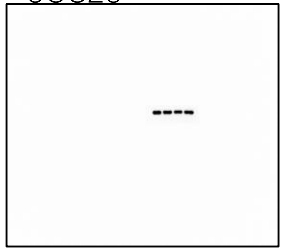

YOD1-SCC25

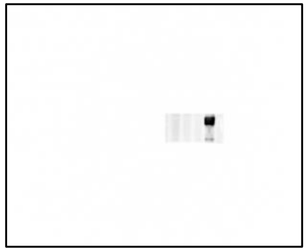

Flag-SCC25

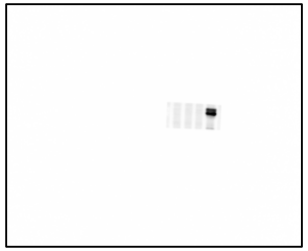

Figure2-E

GAPDH

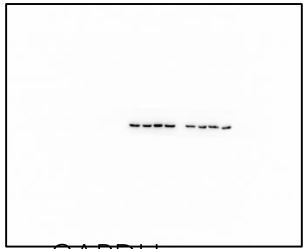

YOD1

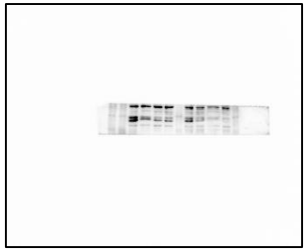

15-GAPDH

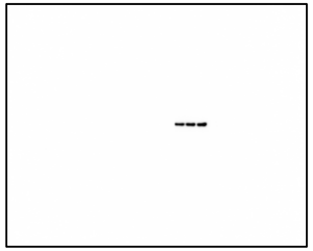

15-YOD1

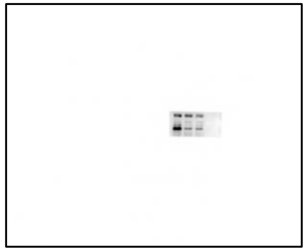

25-GAPDH

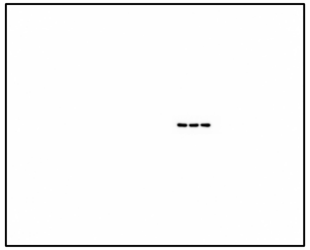

25-YOD1

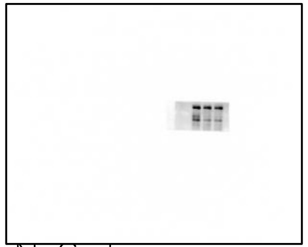

Figure3-E

GAPDH-SCC15

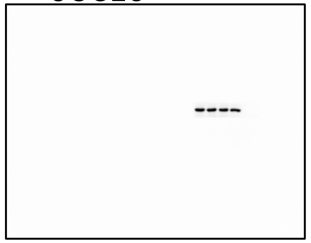

Twist-SCC15

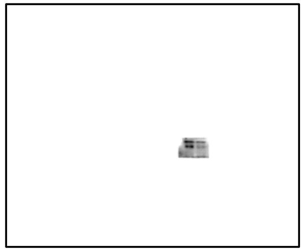

Snail-SCC15

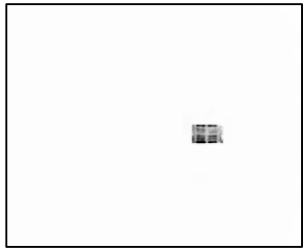

Vimentin-SCC15

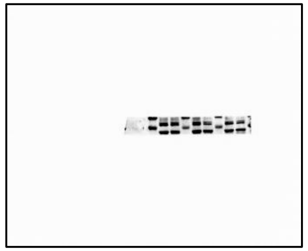

E-Cadnerin-SCC15

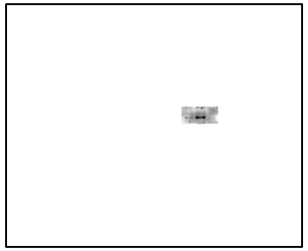

N-Cadnerin-SCC15

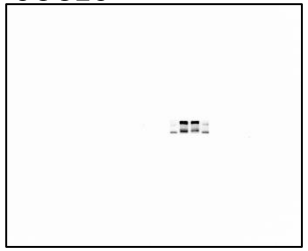

Zeb-1-SCC15

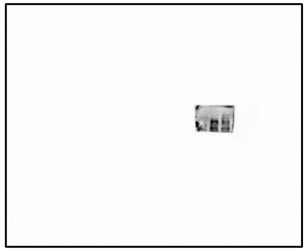

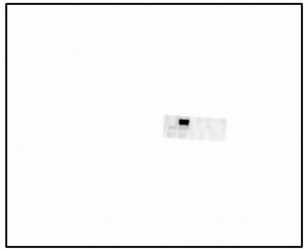

YOD1-SCC15

Figure3-E

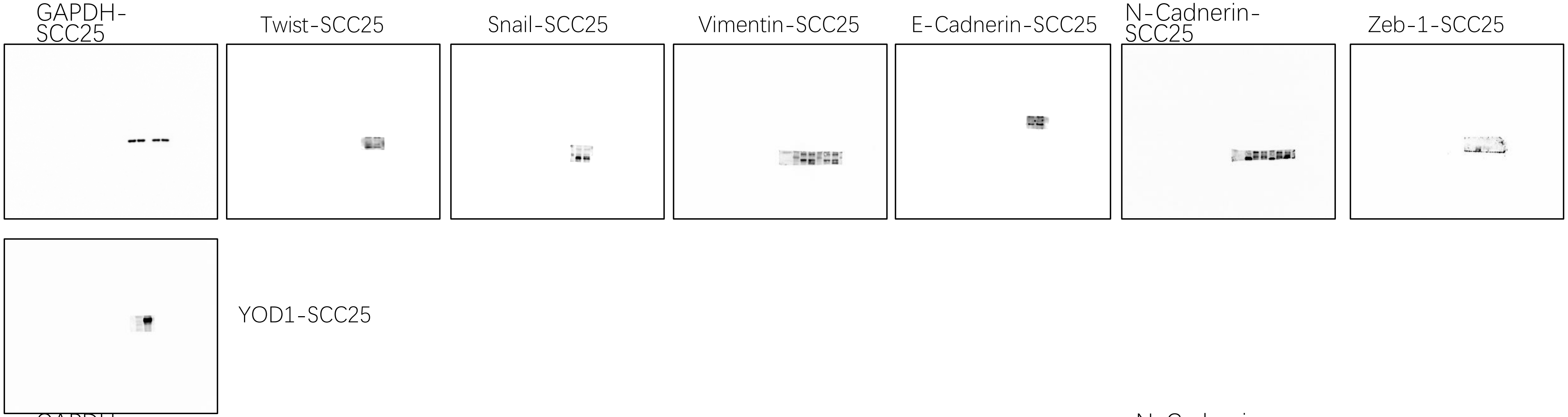

Figure3-F

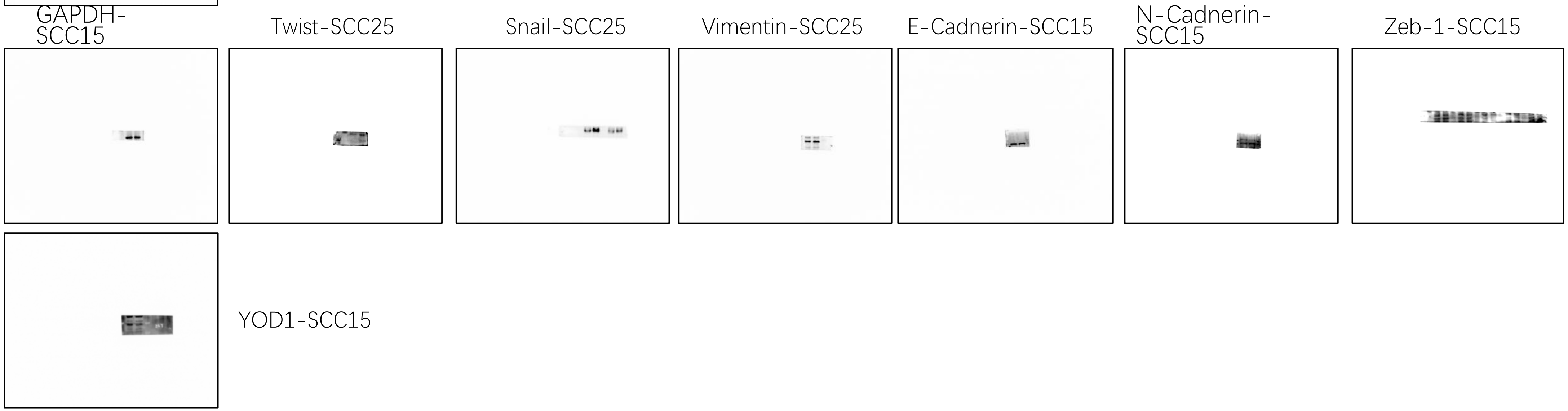

Figure3-F

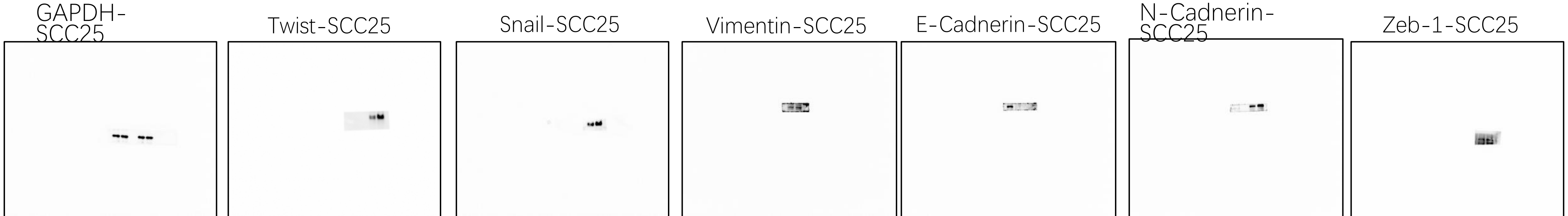

Figure3-F

YOD1-SCC25

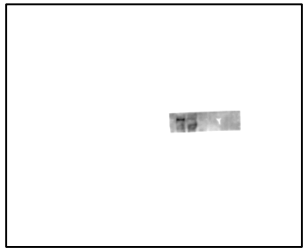

Figure4-B

YOD1-15 TRIM33 YOD1-25

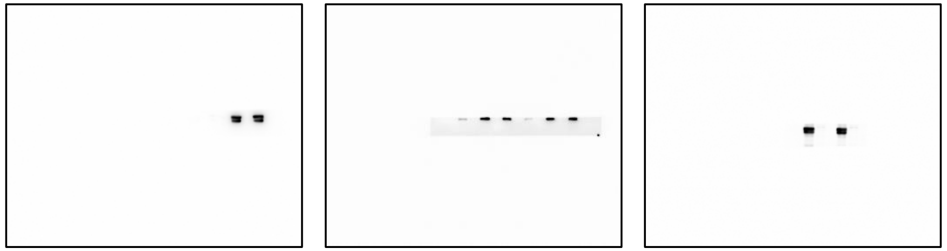

Figure4-D

GAPDH TRIM33

SCC15

TRIM33

SCC25

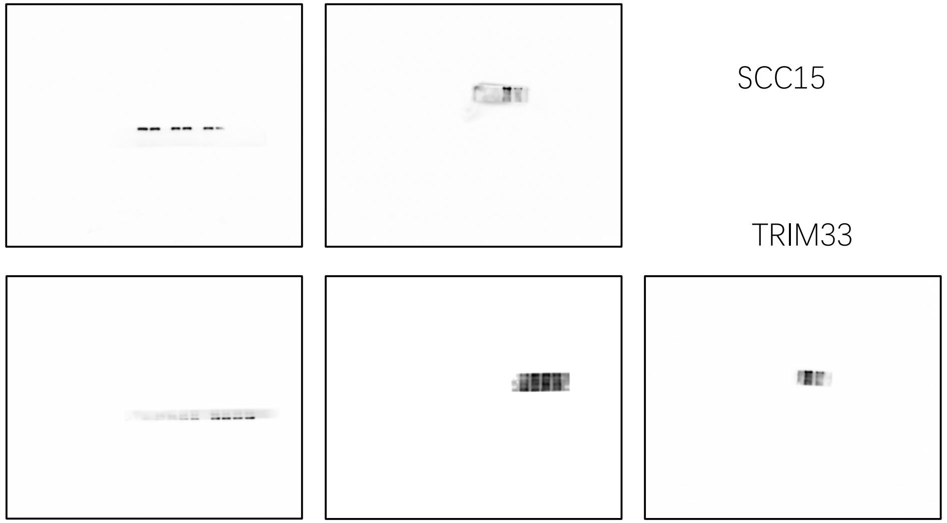

Figure4-E

GAPDH YOD1 TRIM33

SCC15

SCC25

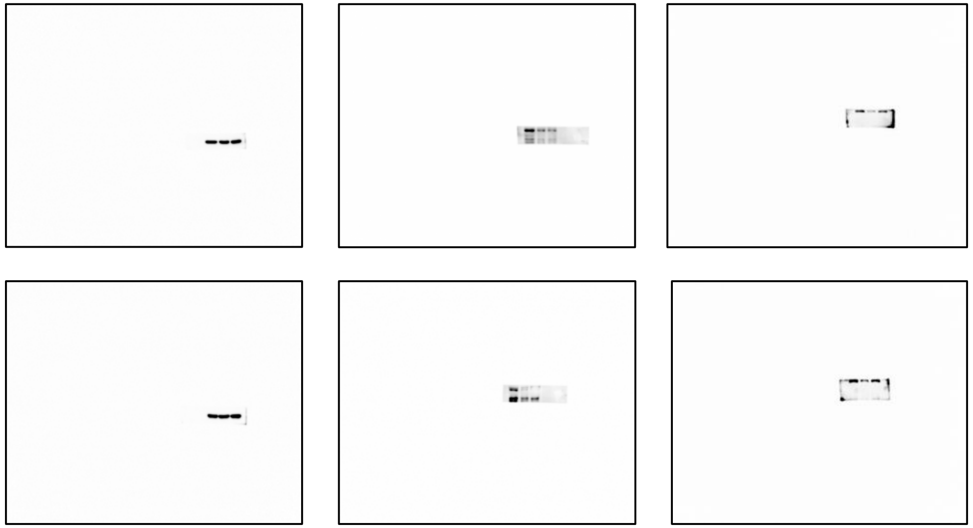

Figure4-F

GAPDH TRIM33

SCC15

SCC25

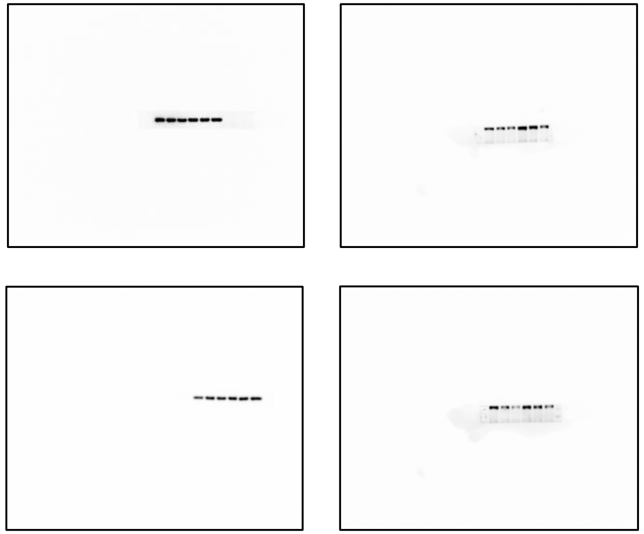

Figure4-G

DAPDH | YOD1 | TRIM33 | IP:TRIM33 | Ub

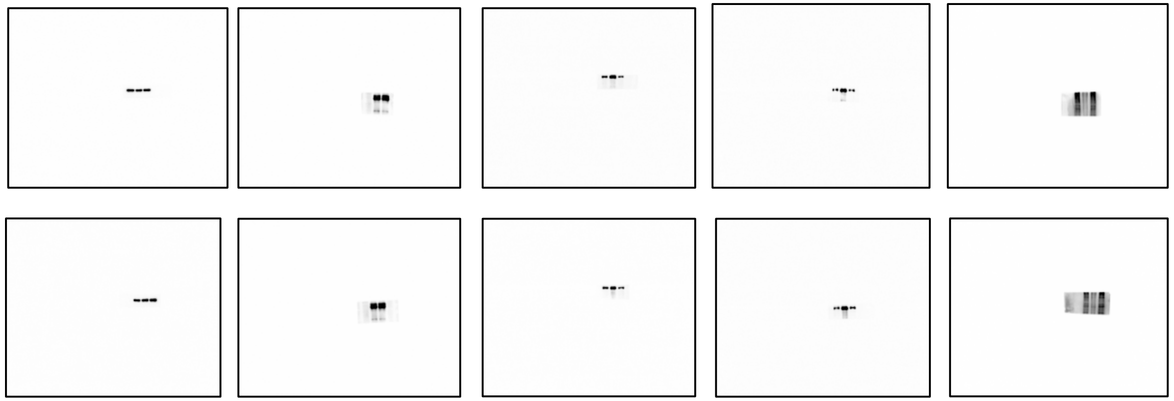

SCC15

SCC25

Figure4-J

GAPDH | T-ERK | P-ERK | T-beta | P-beta

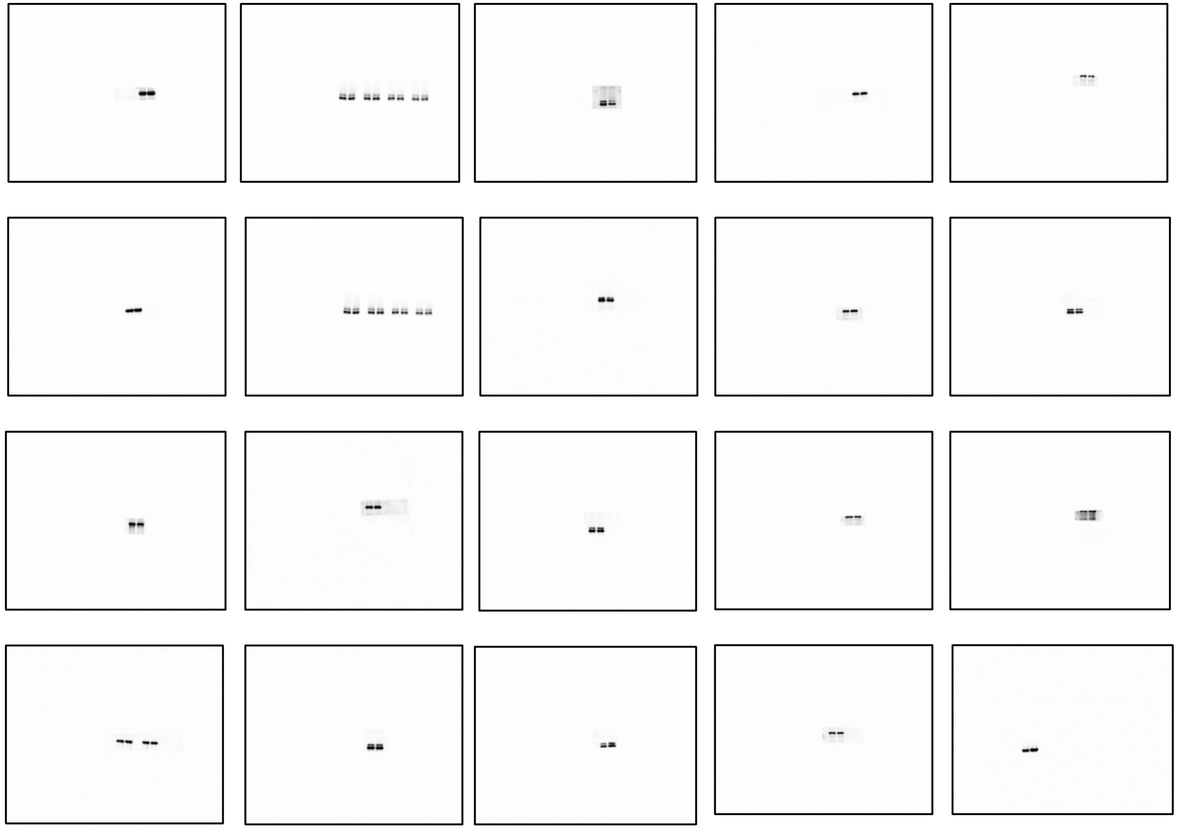

SCC15 — NC

SCC25 — YOD1

SCC15 — si-NC

SCC25 — si-YOD1

Figure5-E

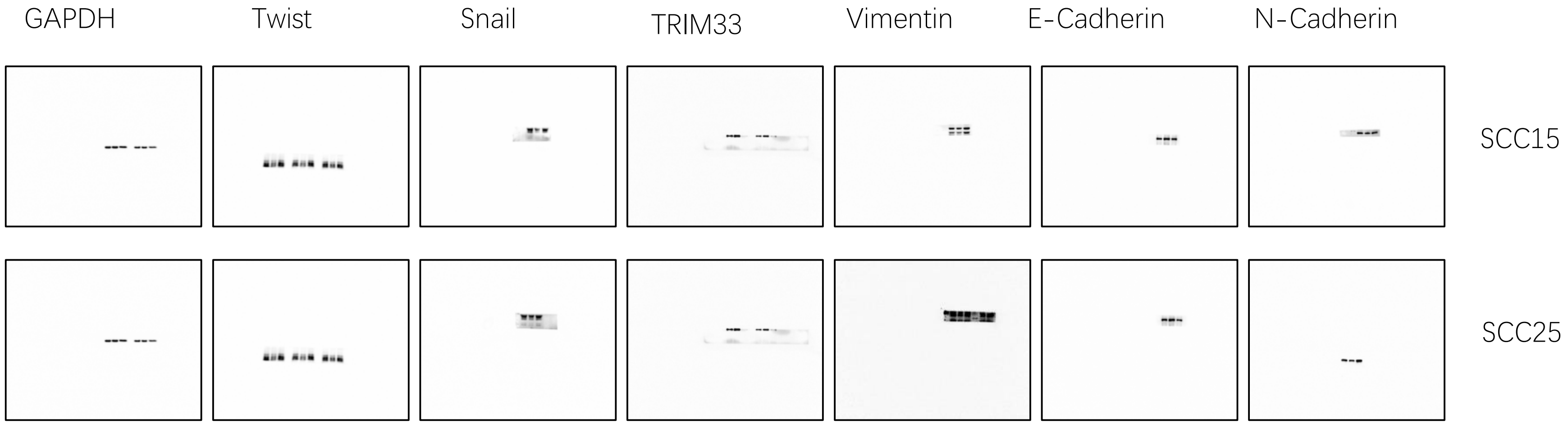

Figure5-F

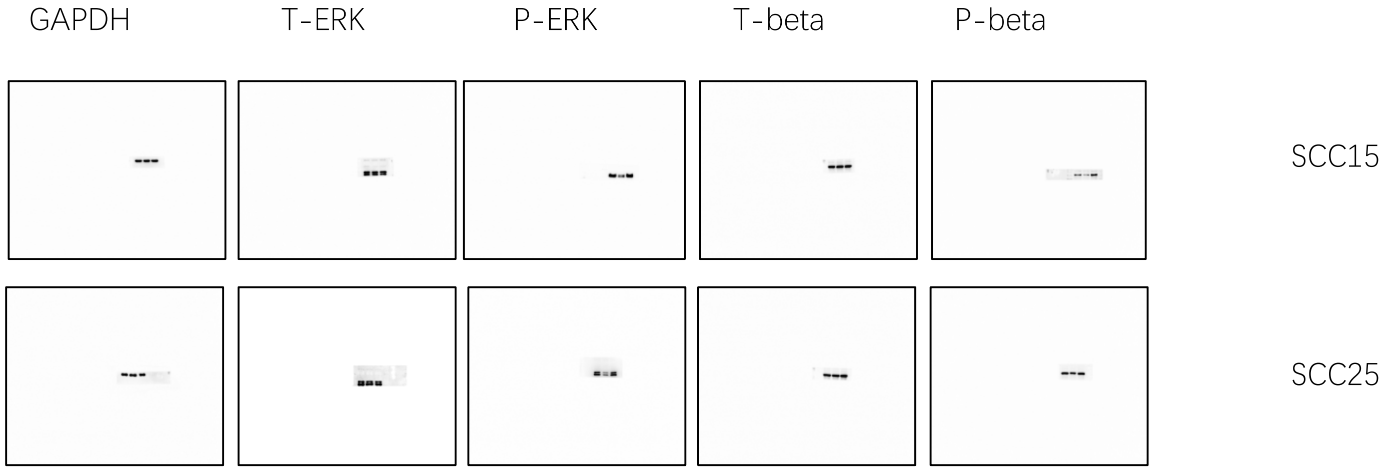

SFigure1

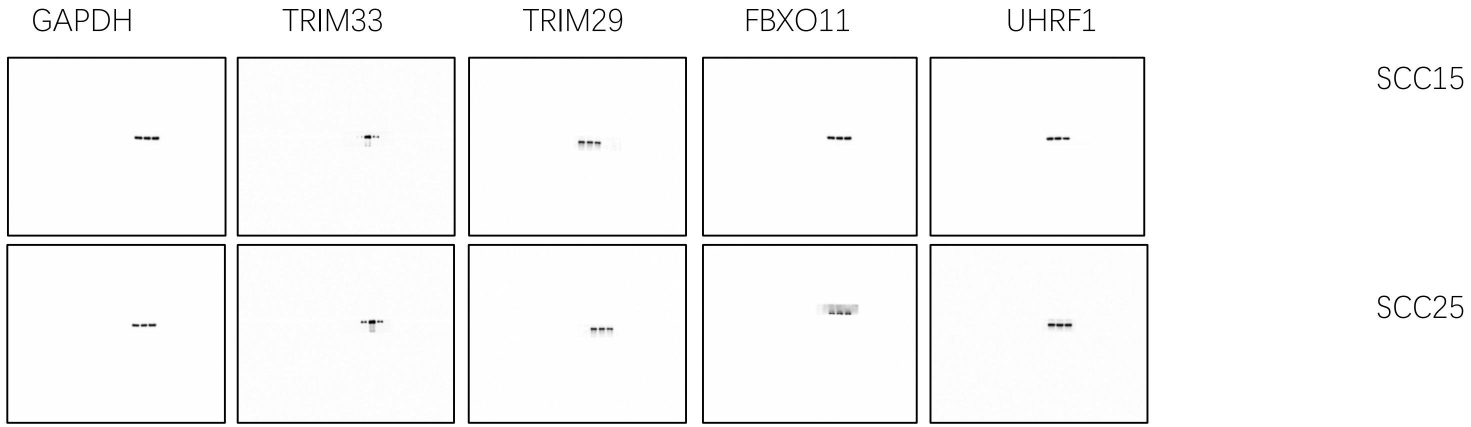

Supplement: Supplementary file 2 — Full uncut gels [file 41419_2023_6035_MOESM2_ESM.pdf]
